# Climate change and health security in Somalia: Building resilient systems for climate-sensitive disease preparedness

**DOI:** 10.1016/j.joclim.2026.100745

**Published:** 2026-09-30

**Authors:** Shukri Hassan Mohamed, Hibo Abdullahi Mohamed, Liban Abdullahi Sudi, Yakub Burhan Abdullahi

**Affiliations:** a Faculty of Health Sciences and Tropical Medicine, Somali National University, Mogadishu, Somalia; b Center for Health Research and Innovation, Somali National University, Mogadishu, Somalia; c Mogadishu Institute of Health, Mogadishu, Somalia; d Faculty of Health Sciences, Salaam university, Mogadishu, Somalia; e Banaadir Research, Consultancy & Evaluation Center (BRCE), Mogadishu, Somalia

**Keywords:** Climate change, Health security, Somalia, Public health preparedness, Health system resilience

Dear Editor

We read with great interest the article by Lokotola et al. entitled “Climate change and primary health care in Africa: A scoping review” published in *The Journal of Climate Change and Health*. The authors highlighted the importance of strengthening climate-resilient primary healthcare systems in Africa. We would like to extend this discussion by emphasizing the need for climate-health preparedness in Somalia [1].

Climate change is rapidly becoming one of the defining health security threats of this century, with effects now visible in the spread of infectious diseases, food insecurity, forced displacement, and health-system strain across vulnerable regions [2,3]. Its consequences are neither distant nor evenly distributed because low-resource settings already facing fragility are bearing the heaviest burden of climate-sensitive illness and disruption [2,4]. Somalia represents a highly vulnerable setting where climate shocks should be understood not only as environmental events but also as contributors to infectious disease risks, social vulnerability, and avoidable health impacts [3].

The public health burden associated with climate change is broad and continues to expand. Across 94 systematic reviews, the most commonly studied climate-related health outcomes were infectious diseases, mortality, and respiratory, cardiovascular, and neurological conditions [2]. Reviews have consistently found that climate change harms human health through extreme weather, higher temperatures, droughts, floods, and related environmental disruption [2]. Infectious diseases are especially important in fragile settings because extreme rain, heat, and flooding can contaminate water, increase mosquito density, and trigger outbreaks that cascade through already stressed communities [3]. Real-world examples from Mozambique and Yemen show how cyclones, stagnant water, poor sanitation, and overcrowded displacement settings can amplify diarrheal disease, malaria, and cholera risk after climate shocks [3].

Somalia is highly exposed to these pathways because climate hazards interact with poverty, malnutrition, displacement, and political instability rather than acting in isolation [3]. Climate-sensitive infectious disease risk is shaped by vulnerability, including biomedical weakness, age, geography, socioeconomic disadvantage, and sociopolitical instability [3]. Such risks are magnified where safeguards are weak, including inadequate sanitation, limited protective infrastructure, and poor capacity to cope and adapt [3]. This is particularly concerning in Africa, where climate change is associated with diverse health burdens and public health systems continue to face substantial challenges due to socioeconomic vulnerabilities [4]. Preparedness remains a central challenge. Although Somalia was not included in the six-country study by Opoku et al., its findings provide relevant regional lessons for climate-health preparedness. Across the six surveyed countries, 63.1% of health professionals reported that climate change had been extensively experienced in recent years, and inadequate infrastructure, medical equipment, emergency response resources, and technical support were identified as barriers to resilience [4]. Respondents also emphasized the need for more hospitals and healthcare centers in hard-to-reach communities, stronger workforce capacity, effective surveillance systems, and coordination between health services and meteorological agencies [4]. These findings offer relevant considerations for Somalia, where climate-health preparedness would benefit from climate-informed surveillance, decentralized preparedness, and resilient primary care that protects displaced families, malnourished children, and other populations least able to absorb repeated shocks [3,4]. Governments, donors, researchers, and global health agencies should prioritize investment in climate-resilient health systems, integrate outbreak preparedness with adaptation policies, and recognize Somalia as an important context for advancing global climate-health preparedness [2,4]. Climate-sensitive infectious diseases require integration into national climate policies and preparedness frameworks, particularly in vulnerable regions such as the Horn of Africa where climate hazards increasingly interact with infectious disease risks [5]. Strengthening climate-resilient health systems requires coordinated adaptation strategies, strengthened healthcare infrastructure, and integration of climate risks into health planning [6].

## Sources of funding

The authors received no financial support for the research, authorship, and/or publication of this article.

## Ethical approval

Ethical approval was not required for this study.

## Consent to participate

Not applicable.

## Consent for publication

Not applicable.

## Availability of data and materials

No new data were generated or analyzed in this study. Therefore, data sharing is not applicable.

## CRediT authorship contribution statement

**Shukri Hassan Mohamed:** Writing – review & editing, Writing – original draft, Conceptualization. **Hibo Abdullahi Mohamed:** Formal analysis. **Liban Abdullahi Sudi:** Validation, Conceptualization. **Yakub Burhan Abdullahi:** Supervision.

## Declaration of competing interest

The authors declare that they have no known competing financial interests or personal relationships that could have appeared to influence the work reported in this paper.
